# Prevalence, risk factors and outcomes of patients coming from the community with sepsis due to multidrug resistant bacteria

**DOI:** 10.1186/s40248-019-0185-4

**Published:** 2019-07-05

**Authors:** Nicolò Capsoni, Pietro Bellone, Stefano Aliberti, Giovanni Sotgiu, Donatella Pavanello, Benedetto Visintin, Elena Callisto, Laura Saderi, Davide Soldini, Luca Lardera, Valter Monzani, Anna Maria Brambilla

**Affiliations:** 1 Department of Emergency Medicine, Fondazione IRCCS Cà Granda Ospedale Maggiore Policlinico, Milan, Italy; 2 Fondazione IRCCS Ca’ Granda Ospedale Maggiore Policlinico, Respiratory Unit and Cystic Fibrosis Adult Center, Via Francesco Sforza 35, 20122 Milan, Italy; 3 Department of Pathophysiology and Transplantation, University of Milan, Milan, Italy; 4 Clinical Epidemiology and Medical Statistics Unit, Department of Biomedical Sciences, Medical Education and Professional Development Unit, AOU Sassari, University of Sassari - Research, Sassari, Italy; 5 Department of Emergency Medicine -ASST-Papa Giovanni XXIII, Bergamo, Italy; 6 Acute Internal Medicine, Fondazione IRCCS Cà Granda Ospedale Maggiore Policlinico, Milan, Italy

**Keywords:** Septic shock, Pneumonia, MRSA, Pseudomonas, ESBL producer *Enterobacteriaceae*

## Abstract

**Background:**

Although previous studies showed an increasing prevalence of infections due to multi-drug resistant (MDR) bacteria in the community, specific data on sepsis are lacking. We aimed to assess prevalence, risk factors and outcomes of patients with sepsis due to MDR bacteria.

**Methods:**

An observational, retrospective study was conducted on consecutive adult patients coming from the community and admitted to the Policlinico Hospital, Milan, Italy, with a diagnosis of sepsis between January 2011 and December 2015. Primary study outcome was in-hospital mortality.

**Results:**

Among 518 patients, at least one MDR bacteria was isolated in 88 (17%). ESBL+ *Enterobacteriaceae* were the most prevalent MDR bacteria (9.7%) followed by MRSA (3.9%). Independent risk factors for sepsis due to MDR bacteria were septic shock (OR: 2.2; *p* = 0.002) and hospitalization in the previous 90 days (OR: 2.3; *p* = 0.003). Independent risk factors for sepsis due to ESBL+ bacteria were hospitalization in the previous 90 days (OR: 2.1; *p* = 0.02) and stroke (OR: 2.1; *p* = 0.04). A significantly higher mortality was detected among patients with vs. without MDR bacteria (40.2% vs. 23.1% respectively, *p* = 0.001). Independent risk factors for mortality among patients with sepsis were coagulation dysfunction (OR: 3.2; *p* = 0.03), septic shock (OR: 3.2; *p* = 0.003), and isolation of a MDR bacteria (OR: 4.6; *p* < 0.001).

**Conclusion:**

In light of the prevalence and impact of MDR bacteria causing sepsis in patients coming from the community, physicians should consider ESBL coverage when starting an empiric antibiotic therapy in patients with specific risk factors, especially in the presence of septic shock.

**Electronic supplementary material:**

The online version of this article (10.1186/s40248-019-0185-4) contains supplementary material, which is available to authorized users.

## Summary

Specific risk factors for MDR bacteria and EBSL+ Enterobacteriaceae are found in a population of 518 patients with sepsis coming from the community. Isolation of a MDR bacteria is associated with higher mortality rates and is an independent risk factor for mortality.

## Introduction

Sepsis is a major health-care problem which affects millions of people each year worldwide with an increasing incidence over the last decades [1]. More than one third of patients with sepsis die during their hospital stay and the economic impact of sepsis is relevant, with a mean and median hospital and intensive care unit (ICU) cost of $32,421 and $27,461 per single patient, respectively [2, 3]. Recently, a new definition of sepsis and an update of the Surviving Sepsis Campaign guidelines have been published to improve sepsis diagnosis, to standardize communication between clinicians and researchers, and to spread an evidence based approach for the management of septic patients [1, 4].

These guidelines confirm the appropriateness of initial antibiotic therapy as a crucial variable in septic patients [1, 5–9]. Among the major contributors of the increasing rates of inappropriate empiric antimicrobial treatment, the spread of antimicrobial resistance seems to be one of the most relevant [5, 6]. An update on sepsis epidemiology due to multi-drug resistant organisms (MDRO) and on its associated risk factors would be very helpful for clinicians to early detect patients requiring a broader antibiotic coverage.

Several experiences reported on risk factors for MDRO infections, enrolling heterogeneous patient populations. Some of them considered infections due to specific MDRO, mainly methicillin-resistant *Staphylococcus aureus* (MRSA) and extended-spectrum beta-lactamase (ESBL) producing *Enterobacteriaceae* [10–15], while others included patients with infection limited to one organ, such as pneumonia and urinary tract infections [16–20]. Finally, few other studies have been published on the impact of bacterial resistances in bloodstream infections, regardless the presence of sepsis [21–23]. No previous studies specifically evaluated prevalence, characteristics, and risk factors for MDRO in a specific sample of patients with sepsis.

The aim of this study was to assess prevalence, characteristics, risk factors, and outcomes of patients suffering of sepsis due to MDRO.

## Materials and methods

### Study design and study patients

This was an observational, retrospective study enrolling consecutive adult patients coming from the community and admitted to the Emergency Department (ED) of the IRCCS Fondazione Ca′ Granda, Ospedale Maggiore Policlinico of Milan, Italy, with sepsis between January 2011 and December 2015. Patients ≥18 years of age fitting sepsis criteria were included [4]. Patients admitted to the ED of the Policlinico Hospital coming from other hospitals were excluded. The study was approved by the ethical committee (262_2017bis) of the IRCCS Fondazione Ca′ Granda, Ospedale Maggiore Policlinico of Milan, Italy, whereas informed consent was waived due to the retrospective and observational nature of the study according to the Italian law on observational studies.

### Data collection

Demographics, comorbidities, risk factors for MDRO, Charlson Comorbidity Index, clinical, laboratory and microbiological findings on admission, site of infection, disease severity (organ dysfunction, septic shock, SOFA score) empiric antibiotic therapy, vasopressor use and invasive mechanical ventilation data were recorded [24, 25].

#### Risk factors for MDRO

The following risk factors for MDRO were recorded: nursing home or extended care facility residency, hospitalization for ≥2 days in the preceding 90 days, antimicrobial therapy in the preceding 90 days, home infusion therapy (including antibiotics), home wound care, indwelling bladder catheter, indwelling intravascular devices, chronic renal failure, chronic dialysis at least during the prior 30 days, day hospital attendance for infusion therapy or blood transfusions, mild, moderate and severe immunodepression [26].

### Study outcomes and definitions

The primary study outcome was in-hospital mortality. Secondary study outcome was length of hospital stay (LOS).

Sepsis and septic shock were defined according to the Third International Consensus Definitions for Sepsis and Septic Shock [4]. Sepsis was defined as life-threatening organ dysfunction caused by a dysregulated host response to infection. Organ dysfunction was identified as an acute change in total SOFA score ≥ 2 points following the infection. Septic shock was defined as a subset of sepsis in which underlying circulatory and cellular metabolism abnormalities are severe enough to increase significantly mortality. This condition was identified with a clinical construct of sepsis with persisting hypotension requiring vasopressors to maintain mean arterial pressure (MAP) ≥65 mmHg and having a serum lactate level > 2 mmol/L (18 mg/dL) despite adequate volume resuscitation.

Severe immunodepression was defined by the presence of at least one of the following medical conditions: active hematologic malignancy, transplantation, immunosuppressive therapy, chemotherapy and radiotherapy in the 30 days before the ED admission. Mild-to-moderate immunodepression was defined by the presence of at least one of the following medical conditions: chronic systemic steroid therapy (prednisone ≥25 mg daily), active solid malignancy, splenectomy, and autoimmune disease.

In-hospital mortality was defined as all-cause mortality occurring during hospitalization. LOS was calculated as the number of days from the date of hospital admission to the date of discharge.

### Microbiological data and empiric antibiotic therapy

Microbiological results performed within 48 h since hospital admission were recorded, including blood, sputum, tracheobronchial aspirate, urine, wound, and mucosal swab cultures, as well as *Legionella pneumophila* and *Streptococcus pneumoniae* urinary antigens. Species identification and antimicrobial susceptibility testing were performed using an automated system (Vitek 2®; BioMérieux); susceptibility break-points were based on Anti-microbial Susceptibility Testing (EUCAST) guidelines. [EUCAST breakpoint table version 1.2 to table version 5.0].

MRSA, *Pseudomonas aeruginosa* resistant to three classes of antibiotics among antipseudomonal penicillins, antipseudomonal cephalosporins, carbapenems, quinolones, and aminoglycosides, vancomycin-resistant *Enterococcus*, *Acinetobacter baumanii*, ESBL producing *Enterobacteriaceae,* carbapenemase-producing *Klebsiella pneumoniae*, and other pathogens with acquired non-susceptibility to at least one agent in three or more antimicrobials categories were considered as MDR bacteria [27].

Empiric antibiotic therapy administered in the ED after diagnosis of sepsis and septic shock was recorded, together with its appropriateness according to the antibiotic susceptibility of the isolated pathogen and the agreement with local guideline (see Additional file 1: Table S1).

#### Statistical analysis

An *ad hoc *electronic form was used to collect all demographic, epidemiological, clinical, and microbiological variables. Qualitative and quantitative data were summarized with absolute and relative (percentage) frequencies and medians (interquartile ranges, IQR) according to their non-parametric distribution, respectively. Statistical differences of qualitative and quantitative variables were assessed with chi-squared or Fisher exact, when appropriate, and Mann-Whitney tests, respectively. Logistic regression analyses were carried out to assess the relationship between MDRO or ESBL+ infection and the collected covariates. A two-tailed *p* less than 0.05 was considered statistically significant. The statistical software STATA 14 (StataCorp LP, Lakeway Drive, College Station, USA) was used to perform all statistical computations.

## Results

### Study population

A total of 663 consecutive patients with sepsis were enrolled during the study period (56.6% males, median [IQR] age: 80 [71–87] years). The final study population consisted of 518 patients (78.1% of the total enrolled) who underwent at least one bacteriological test within the first 24 h after hospitalization.

Demographics, comorbidities, risk factors for MDRO, clinical, and laboratory findings on admission, site of infection, disease severity, therapy prescribed within 24 h after admission are summarized in Table 1: lung (59.9%) and urinary tract (36.4%) were the most common sites of infection. A total of 146 (28.2%) patients had septic shock.

**Table 1:** Study population. (518)

Demographics characteristics	
*Female, n (%)*	220 (42.5)
*Median (IQR) age, years*	80 (71–87)
Comorbidities, *n (%)*	
*Median (IQR) Charlson comorbidity index*	6 (5–8)
*Hypertension*	289 (55.8)
*Diabetes mellitus*	124 (23.9)
*Ischemic heart disease*	109 (21.0)
*COPD*	114 (22.0)
*Stroke*	83 (16.0)
*Dementia*	79 (15.3)
*Chronic heart failure*	50 (9.7)
*Chronic liver disease*	38 (7.3)
*Peripheral vascular disease*	28 (5.4)
*Hemiplegia*	21 (4.1)
*Cirrhosis*	25 (4.8)
Risk factors for MDR pathogens, *n (%)*	
*Patients with at least one risk factor for MDR pathogens*	397 (76.6)
*Hospitalization in the past 90 days*	147 (28.4)
*Median (IQR) LOS*	13 (8–21)
*Day hospital attendance in the past 90 days*	100 (19.3)
*Antibiotic therapy in the past 90 days*	80 (15.5)
*Severe immunosuppression*	98 (18.9)
*Mild /moderate immunosuppression*	83 (16.0)
*Solid cancer*	73 (14.1)
*Chronic steroid therapy*	68 (13.1)
*Haematological malignancy*	45 (8.7)
*Chemotherapy*	28 (5.4)
*AIDS*	3 (0.6)
*Chronic renal failure*	96 (18.5)
*Dialysis*	16 (3.1)
*Home wound care/infusion therapy*	82 (15.8)
*Indwelling bladder catheter*	70 (13.5)
*Nursing home or LTCF residency*	45 (8.7)
*Indwelling intravascular catheters*	31 (6.0)
Clinical findings	
*Median (IQR) body temperature, °C*	38.0 (37.1–38.7)
*Median (IQR) systolic blood pressure, mmHg*	110 (90–135)
*Median (IQR) diastolic blood pressure, mmHg*	60 (50–70)
*Median (IQR) mean blood pressure, mmHg*	77 (63–92)
*Median (IQR) heart rate, bpm*	102 (88–120)
*Median (IQR) oxygen saturation, %*	95 (91–97)
*Median (IQR) respiratory rate, bpm*	22 (18–30)
*Median (IQR) shock index*	0.9 (0.7–1.2)
Laboratory findings	
*Median (IQR) arterial pH*	7.5 (7.4–7.5)
*Median (IQR) PaCO*_*2*_*, mmHg*	30 (25–35)
*Median (IQR) PaO*_*2*_*, mmHg*	65 (55–77)
*Median (IQR) HCO*_*3*_^*−*^ *mEq/L*	21.8 (18.0–24.6)
*Median (IQR) PaO*_*2*_*/FiO*_*2*_ *ratio*	276 (229–333)
*Median (IQR) lactate, mEq/L*	2.9 (2.1–4.3)
*Median (IQR) white blood cells, cell/L*^*−1*^	12.1 (7.3–17.9)
*Median (IQR) platelets, cell/L*^*−1*^	190.0 (131.0–258.5)
*Mean (SD) haemoglobin, g/dL*	12.3 (2.3)
*Median (IQR) glucose, mg/dL*	141 (110–192)
*Median (IQR) urea, mg/dL*	65 (46–97)
*Median (IQR) creatinine, mg/dL*	1.6 (1.2–2.4)
*Median (IQR) C-reactive protein, g/dL*	11.4 (4.5–22.7)
*Median (IQR) aspartate aminotransferase U/l*	28 (20–50)
*Median (IQR) alanine aminotransferase U/l*	21 (13–39)
*Median (IQR) lactate dehydrogenase*	327 (222–457)
*Median (IQR) total bilirubin, mg/dL*	0.8 (0.5–1.6)
*Median (IQR) INR*	1.3 (1.2–1.5)
Site of primary infection*, n (%)*	
*Lung*	273 (59.9)
*Urinary tract*	166 (36.4)
*Abdomen*	59 (12.9)
*Skin and soft tissue*	31 (6.8)
*Central nervous system*	9 (2.0)
*Bone and joints*	4 (0.9)
*More than one site*	82 (15.8)
*Unknown origin*	62 (12.0)
MOF*, n (%)*	
*Metabolic dysfunction*	359 (75.6)
*Renal failure*	223 (43.6)
*Hemodynamic failure*	200 (39.0)
*Cognitive impairment*	158 (32.9)
*Shock*	146 (28.2)
*Respiratory failure*	94 (18.4)
*Liver failure*	67 (13.9)
*Coagulation dysfunction*	56 (12.1)
*Haematological dysfunction*	51 (9.9)
Antibiotics*, n (%)*	
*Azithromycin*	40 (5.5)
*Piperacillin/Tazobactam*	188 (25.9)
*Ceftriaxone*	164 (22.6)
*Levofloxacin*	111 (15.3)
*Imipenem*	66 (9.1)
*Vancomycin*	51 (7.0)
*Ciprofloxacin*	28 (3.9)
*Amoxicillin/Clavulanate*	22 (3.0)
*Metronidazole*	16 (2.2)
*Meropenem*	5 (0.7)
*Ampicillin*	7 (1.0)
*Amikacin*	7 (1.0)
*Ceftazidime*	5 (0.7)
*Others*	16 (2.2)
*Appropriate empiric antibiotic therapy according to local guidelines*	273 (58.3)
*Appropriate empiric antibiotic therapy according to antibiotic susceptibility of the isolated pathogen*	155 (66.8)
*Use of vasopressors*	84 (16.2)
*Mechanical ventilation*	8 (2.5)

*n* number, *IQR* interquartile range, *COPD* chronic obstructive pulmonary disease, *AIDS* Acquired immune deficiency syndrome; *LTCF* long term care facility, *INR* International normalized ratio; MOF: multi organ failure (other than primary site of infection)

Three hundred ninety-seven patients (76.6%) had at least one risk factor for MDRO: hospitalization in the previous 3 months (28.4%), day hospital attendance (19.3%), severe immunodepression (18.9%), and antibiotic therapy in the last 90 days (15.5%) were the most frequent risk factors for MDRO.

### Prevalence and characteristics of patients with sepsis due to MDRO and ESBL+ bacteria

Cultures were performed on blood (*n* = 446, 86.1%) and sputum (*n* = 25, 4.8%) samples, tracheobronchial aspirates (*n* = 27, 5.2%), urine (*n* = 189, 36%] and other (*n* = 47, 9.1%) samples. Microbiological findings are summarized in Table 2. At least one pathogen was isolated in 305 patients (58.9%) and, among them, 198 (44.5%) had bacteremia.

**Table 2:** Microbiological findings

Microbiological*, n (%)*	
*Patients with isolated microorganism*	305 (58.9)
*Patients with at least one MDR organism*	88 (17.0)
*Patients with at least one ESBL organism*	50 (9.7)
*E. coli ESBL +*	43 (8.3)
*Methicillin-resistant S. aureus*	20 (3.9)
*Proteus spp MDR +*	5 (1)
*K. pneumoniae ESBL +*	7 (1.4)
*K. pneumomiae carbapenemase producer*	6 (1.2)
*P. aeruginosa MDR+*	4 (0.8)
*Enterococcus spp MDR+*	2 (0.4)
*Enterobacter spp MDR +*	2 (0.4)
*Stenotrophomonas maltophilia MDR+*	1 (0.2)
*E. coli ESBL -*	77 (14.9)
*Methicillin-sensible S. aureus*	24 (4.6)
*S. pneumoniae*	24 (4.6)
*K. pneumoniae ESBL-*	17 (3.3)
*P. aeruginosa MDR-*	25 (4.8)
*Candida spp*	12 (2.3)
*Enterococcus spp MDR-*	10 (1.9)
*Proteus spp MDR-)*	9 (1.7)
*Enterobacter spp MDR-*	5 (1)
*N. meningitides*	3 (0.6)
*Bacteroides spp*	1 (0.2)
*Providencia spp*	2 (0.4)
*Stenotrophomonas maltophilia MDR-*	3 (0.6)
*C. difficile*	4 (0.8)
*Aspergillus spp*	2 (0.4)
*Acinetobacter baumanii*	1 (0.2)
*Salmonella group B*	2 (0.4)
*H. influenzae*	1 (0.2)
*Acinetobacter iwoffii*	1 (0.2)
*Propiniobacterium*	1 (0.2)
*Serratia spp*	2 (0.4)
*Polymicrobial infection*	29 (5.6)

*n* number, *ESBL* extended spectrum beta lactamase, *MDR* multi-drug resistant, *spp* species

At least one MDRO was isolated in 88 patients (17% among the entire population and 29.1% among culture positive patients), with ESBL+ *Enterobacteriaceae* being the most prevalent isolates (50 patients, 9.7%), including 43 (8.3%) patients with ESBL+ *E. coli* and 7 (1.4%) with ESBL+ *K. pneumoniae*. The second most prevalent MDR bacteria were MRSA (20 patients, 3.9%).

Demographics, comorbidities, risk factors, clinical and laboratory findings on admission, site of infection, disease severity, appropriateness of empiric antibiotic therapy, vasopressor use and invasive mechanical ventilation of the study sample are reported in the online supplement according to the presence of MDRO (Additional file 1: Table S2) and ESBL+ bacteria (Additional file 1: Table S3).

### Independent risk factors associated to MDRO and ESBL+ infection in patients with sepsis

After adjusting for several confounders, independent risk factors associated with the occurrence of sepsis due to MDRO and ESBL+ bacteria are reported in Tables 3 and 4. Significant independent risk factors for sepsis due to MDRO were septic shock (OR: 2.2; 95% CI: 1.3–3.7, *p* = 0.002) and hospitalization in the past 90 days (OR: 2.3; 95% CI: 1.3–4.1, *p* = 0.003). Significant independent risk factors for sepsis due to ESBL+ bacteria were hospitalization in the past 90 days (OR: 2.1; 95% CI: 1.2–3.9, *p* = 0.02) and stroke (OR: 2.1; 95% CI: 1.0–4.1, *p* = 0.04).

**Table 3:** Logistic regression analysis to assess the relationship between MDR infection and demographic, epidemiological, clinical, and laboratory variables. (518)

Variables	Univariate analysis		Multivariate analysis	
	OR (95% CI)	*p*	OR (95% CI)	*p*
*Female*	1.2 (0.8–1.9)	0.39	1.2 (0.7–2.0)	0.42
*Age*	1.0 (1.0–1.0)	0.62	1.0 (1.0–2.0)	0.93
*Septic shock*	2.1 (1.3–3.4)	0.002	2.2 (1.3–3.7)	0.002
*Antibiotic therapy in the past 90 days*	1.7 (1.1–3.3)	0.03	1.0 (0.5–2.0)	1.0
*Hospitalization in the past 90 days*	3.2 (2.0–5.1)	< 0.0001	2.3 (1.3–4.1)	0.003
*Indwelling bladder catheter*	2.2 (1.3–4.0)	0.006	1.9 (0.9–3.9)	0.11
*Home wound care/infusion therapy*	2.1 (1.2–3.6)	0.01	1.4 (0.7–2.8)	0.39
*Chronic heart failure*	2.1 (1.1–4.0)	0.03	1.7 (0.8–3.6)	0.14
*Peripheral vascular disease*	0.8 (0.3–2.4)	0.70		
*Stroke*	2.0 (1.2–3.5)	0.01	1.8 (0.9–3.3)	0.08
*Dementia*	1.9 (1.1–3.3)	0.03	1.6 (0.8–3.0)	0.17
*Haematological malignancy*	2.2 (1.1–4.3)	0.03	1.6 (0.7–3.6)	0.28
*Chronic steroid therapy*	2.1 (1.2–3.9)	0.01	1.6 (0.8–3.2)	0.19

**Table 4:** Logistic regression analysis to assess the relationship between ESBL infection and demographic, epidemiological, clinical, and laboratory variables. (518)

Variables	Univariate analysis		Multivariate analysis	
	OR (95% CI)	*p*	OR (95% CI)	*p*
*Female*	1.0 (0.5–1.8)	0.94	1.0 (0.5–1.8)	0.87
*Age*	1.0 (1.0–1.1)	0.10	1.0 (1.0–1.0)	0.50
*Hospitalization in the past 90 days*	2.4 (1.3–4.3)	0.004	2.1 (1.2–3.9)	0.02
*Indwelling bladder catheter*	2.5 (1.3–5.1)	0.008	2.0 (0.9–4.1)	0.08
*Chronic heart failure*	2.3 (1.0–5.0)	0.04	1.8 (0.8–4.1)	0.18
*Stroke*	2.5 (1.3–4.9)	0.006	2.1 (1.0–4.1)	0.04
*Dementia*	2.4 (1.2–4.7)	0.01	2.0 (1.0–4.0)	0.06

### Study outcomes

In-hospital mortality was 25.7% (*n* = 133). Among patients with septic shock 69 (47.3%) died. Demographics, comorbidities, risk factors, clinical laboratory, and microbiological findings on admission, site of infection, disease severity, appropriateness of empiric antibiotic therapy, vasopressors use and invasive mechanical ventilation of patients who died versus those who survived are summarized in Table 5.

**Table 5:** Study population according to in-hospital mortality. (518)

Variable	Survivors	Death	*p*
*Hospital mortality, n (%)*	378 (74.0)	133 (25.7)	–
Demographics characteristics			
*Female, n (%)*	158 (41.8)	59 (44.4)	0.61
*Median (IQR) age*	78 (69–85)	81 (75–87)	0.01
Comorbidities*, n (%)*			
*COPD*	85 (22.5)	28 (21.1)	0.73
*Diabetes mellitus*	99 (26.2)	23 (17.3)	0.04
*Hypertension*	211 (55.8)	75 (56.4)	0.91
*Ischemic heart disease*	73 (19.3)	36 (27.1)	0.06
*Chronic heart failure*	35 (9.3)	15 (11.3)	0.50
*Peripheral vascular disease*	14 (3.7)	14 (10.5)	0.003
*Stroke*	54 (14.3)	27 (20.3)	0.10
*Hemiplegia*	14 (3.7)	7 (5.3)	0.44
*Dementia*	54 (14.3)	24 (18.1)	0.30
*Chronic liver disease*	27 (7.1)	11 (8.3)	0.67
*Cirrhosis*	16 (4.2)	8 (6.0)	0.40
*Chronic renal failure*	64 (16.9)	31 (23.3)	0.10
*Active dialysis*	12 (3.2)	4 (3.0)	1.0
*Solid cancer*	53 (14.0)	20 (15.0)	0.77
*Haematological malignancy*	28 (7.4)	16 (12.0)	0.10
*AIDS*	2 (0.5)	1 (0.8)	1.0
*Chemotherapy*	19 (5.0)	9 (6.8)	0.45
*Severe immunosuppression*	62 (16.4)	34 (25.6)	0.02
*Mild/moderate immunosuppression*	61 (16.1)	22 (16.5)	0.91
*Chronic steroid therapy*	46 (12.2)	22 (16.5)	0.20
*Median (IQR) Charlson comorbidity index*	6 (4–8)	7 (5–9)	0.001
Risk factors*, n (%)*			
*LTCF*	27 (7.1)	18 (13.5)	0.03
*Antibiotic therapy in the past 90 days*	50 (13.3)	30 (22.9)	0.009
*Hospitalization in the past 90 days*	91 (24.1)	55 (41.4)	< 0.0001
*Home wound care/infusion therapy*	56 (14.8)	25 (18.8)	0.28
*Day hospital attendance*	73 (19.3)	25 (18.8)	0.90
*Indwelling bladder catheter*	49 (12.9)	20 (15.0)	0.55
*Indwelling intravascular catheters*	24 (6.4)	7 (5.3)	0.65
Clinical findings			
*Median (IQR) body temperature, °C*	38.0 (37.2–38.8)	37.6 (36.6–38.5)	0.03
*Median (IQR) systolic blood pressure, mmHg*	113 (90–140)	99 (80–120)	0.0001
*Median (IQR) diastolic blood pressure, mmHg*	60 (50–73)	55 (46–68)	0.0006
*Median (IQR) mean blood pressure, mmHg*	78 (63–93)	70 (60–83)	0.0001
*Median (IQR) heart rate, bpm*	102 (87–120)	103 (88–120)	0.99
*Median (IQR) oxygen saturation, %*	95 (92–98)	94 (90–97)	0.06
*Median (IQR) respiratory rate, bpm*	22 (18–28)	28 (18–35)	0.008
*Median (IQR) shock index*	0.9 (0.7–1.2)	1.0 (0.8–1.3)	0.002
Laboratory findings			
*Median (IQR) arterial pH*	7.5 (7.4–7.5)	7.4 (7.4–7.5)	0.0004
*Median (IQR) PaCO*_*2*_*, mmHg*	30.0 (26.0–35.0)	31.0 (24.0–36.5-)	0.80
*Median (IQR) PaO*_*2*_*, mmHg*	65 (55–76)	67 (55–82)	0.27
*Median (IQR) HCO*_*3*_*- mEq/L*	22.0 (18.3–25.0)	20.7 (15.4–24.0)	0.009
*Median (IQR) PaO*_*2*_*/FiO*_*2*_ *ratio*	281.0 (233.0–333.0)	271.5 (222.0–335.5)	0.88
*Median (IQR) lactate, mEq/L*	2.8 (2.1–4.0)	3.1 (2.1–5.7)	0.02
*Median (IQR) white blood cells, cell/L*^*−1*^	12.2 (7.4–17.8)	12.5 (7.3–19.3)	0.50
*Median (IQR) platelet, cell/L*^*− 1*^	198.0 (140.0–254.0)	181.0 (105.5–276.0)	0.45
*Mean (SD) haemoglobin, g/dL*	12.6 (2.2)	11.4 (2.3)	< 0.0001
*Median (IQR) glucose, mg/dL*	144.5 (113.0–198.0)	126.5 (100.5–185.0)	0.004
*Median (IQR) urea, mg/dL*	58 (43–87)	87 (63–145)	< 0.0001
*Median (IQR) creatinine, mg/dL*	1.5 (1.1–2.1)	2.0 (1.5–4.1)	< 0.0001
*Median (IQR) C-reactive protein, g/dL*	10.0 (3.8–21.6)	15.7 (7.1–25.9)	0.0006
*Median (IQR) Aspartate aminotransferase*	28 (20–46)	33 (20–65)	0.05
*Median (IQR) Alanine aminotransferase*	21 (13–40)	24 (12–39)	0.85
*Median (IQR) total bilirubin, mg/dL*	0.8 (0.5–1.7)	1.0 (0.6–1.6)	0.52
*Median (IQR) INR*	1.3 (1.1–1.5)	1.3 (1.2–1.7)	0.40
Site of infection, *n (%)*			
*Lung*	192 (57.3)	77 (67.0)	0.07
*Urinary tract*	134 (40.0)	31 (27.0)	0.01
*Central nervous system*	6 (1.8)	2 (1.7)	1.0
*Abdomen*	43 (12.8)	16 (13.9)	0.77
*Skin and soft tissue)*	18 (5.4)	13 (11.3)	0.03
*Bone and joints*	3 (0.9)	1 (0.9)	1.0
*Multiple origin*	58 (15.3)	24 (18.1)	0.47
*Unknown origin*	43 (11.4)	18 (13.5)	0.51
Severity of disease*, n (%)*			
*Hemodynamic failure*	136 (36.3)	64 (48.9)	0.01
*Respiratory failure*	71 (19.0)	23 (17.6)	0.71
*Renal failure)*	153 (41.1)	68 (51.5)	0.04
*Liver failure*	44 (12.6)	23 (18.1)	0.12
*Cognitive impairment*	106 (29.9)	49 (40.8)	0.03
*Haematological dysfunction*	34 (9.0)	15 (11.5)	0.42
*Coagulation dysfunction*	33 (9.9)	22 (18.0)	0.02
*Metabolic dysfunction*	261 (74.8)	94 (78.3)	0.43
*Shock*	75 (19.8)	69 (51.9)	< 0.0001
Microbiological findings*, n (%)*			
*Blood cultures performed in the first 48 h*	326 (86.2)	114 (85.7)	0.88
*Bacteremia*	148 (45.4)	48 (42.1)	0.54
*Cultures*	()	()	
*Culture positive*	224 (59.3)	77 (57.9)	0.78
*Polymicrobial infection*	16 (7.1)	13 (16.7)	0.01
*MDR pathogen isolated*	52 (13.8)	35 (26.3)	0.001
*ESBL producer pathogen isolated*	33 (8.7)	16 (12.0)	0.27
*MRSA isolated*	6 (1.6)	14 (10.5)	< 0.0001
*Appropriate empiric antibiotic therapy according to local guidelines*	190 (56.6)	77 (61.6)	0.33
*Appropriate empiric antibiotic therapy according to antibiotic susceptibility of the isolated pathogen*	118 (71,1%)	56 (37,1%)	0,058
*Use of vasopressors*	49 (13.0)	35 (26.3)	< 0.0001
*Mechanical ventilation*	4 (1.6)	4 (5.3)	0.09

*n* number, *IQR* interquartile range, *COPD* chronic obstructive pulmonary disease, *AIDS* Acquired immune deficiency syndrome, *LTCF* long term care facility, *INR* International normalized ratio, *MOF* multi organ failure (other than primary site of infection)

The median (IQR) LOS was 13 (8–21) days. Among patients with MDRO the median (IQR) LOS was 15 (9–22) days and in-hospital mortality was 40.2% (35 patients), while among those without MDRO infection LOS was 13 (8–21) days (*p* = 0.36) and mortality was 23.1% (*n* = 98) (*p* = 0.001).

Among patients with ESBL+ infection the median (IQR) LOS was 15 (9–21) days and in-hospital mortality was 32% (*n* = 16), whereas among those without ESBL+ infection median (IQR) LOS was 13 (8–22) days (*p* = 0.73) and in-hospital mortality was 25.3% (*n* = 117) (*p* = 0.27).

### Risk factors for mortality in patients with sepsis

After adjusting for several confounders, including antibiotic therapy, vasopressor exposure and ventilatory treatment, independent risk factors associated with in-hospital mortality in patients with sepsis were: coagulation dysfunction (OR: 3.2; 95% CI: 1.1–8.8, *p* = 0.03), septic shock (OR: 3.2; 95% CI: 1.5–7.0, *p* = 0.003), and isolation of a MDR pathogen (OR: 4.6; 95% CI: 2.0–10.6, *p* < 0.001) (Table 6).

**Table 6:** Logistic regression analysis to assess the relationship between hospital mortality and demographic, epidemiological, clinical, and laboratory variables. (518)

Variables	Univariate analysis		Multivariate analysis	
	OR (95% CI)	*p*	OR (95% CI)	*p*
*Female*	1.1 (0.7–1.7)	0.61	1.1 (0.5–2.3)	0.79
*Age*	1.0 (1.0–1.0)	0.01	1.0 (1.0–1.1)	0.053
*Septic shock*	4.4 (2.9–6.7)	< 0.0001	3.2 (1.5–7.0)	0.003
*Nursing home*	2.0 (1.1–3.8)	0.03	0.4 (0.1–1.4)	0.14
*Antibiotic therapy in the past 90 days*	1.9 (1.2–3.2)	0.01	1.1 (0.4–3.0)	0.90
*Hospitalization in the past 90 days*	2.2 (1.5–3.4)	< 0.0001	1.1 (0.4–2.7)	0.85
*Diabetes mellitus*	0.6 (0.4–1.0)	0.04	0.8 (0.3–2.1)	0.71
*Peripheral vascular disease*	3.1 (1.4–6.6)	0.004	1.4 (0.3–5.9)	0.67
*Severe immunosoppression*	1.8 (1.1–2.8)	0.02	1.9 (0.8–4.9)	0.17
*Lung site*	1.5 (1.0–2.4)	0.07	1.7 (0.7–4.0)	0.21
*Urinary tract site*	0.6 (0.4–0.9)	0.01	0.6 (0.3–1.5)	0.27
*Skin and soft tissue site*	2.2 (1.1–4.7)	0.03	3.2 (0.9–11.4)	0.07
*Renal failure*	1.5 (1.0–2.3)	0.04	1.5 (0.7–3.1)	0.26
*Cognitive impairment*	1.6 (1.1–2.5)	0.03	1.5 (0.7–3.0)	0.30
*Coagulation dysfunction*	2.0 (1.1–3.6)	0.02	3.2 (1.1–8.8)	0.03
*SOFA*	1.3 (1.2–1.4)	< 0.0001		
*Quick SOFA*	1.5 (1.1–2.1)	0.008		
*MDR pathogen isolated*	2.2 (1.4–3.6)	0.001	4.6 (2.0–10.6)	< 0.001
*Use of vasopressors*	2.4 (1.5–3.9)	< 0.0001		
*Polymicrobial infection*	2.6 (1.2–5.7)	0.02	2.4 (0.7–7.7)	0.15

## Discussion

The present study shows that more than three quarters of patients admitted to the hospital from the community for sepsis have at least one risk factor for MDRO, while in 17% of patients a MDRO is isolated. Among those, ESBL+ *Enterobacteriaceae* are the most prevalent ones (9.7%). Hospitalization in the previous 90 days and the presence of septic shock are the two independent risk factors associated with MDRO in patients with sepsis, whereas hospitalization in the previous 90 days and stroke are independently associated with infection caused by ESBL producer *Enterobacteraceae*.

Our study is in line with previously published data in terms of frequency of different sites of infection (lung being the first followed by urinary tract), most frequent organs involved in multi-organ failure, percentage of patients with septic shock and mortality rate [28, 29]. The prevalence of MDRO in our study is slightly higher than those previously reported in literature, mainly because of the characteristics of our study sample characterized by elderly patients with several comorbidities and a long history of medicalization [5, 30, 31]. We found a discrepancy between the frequency of risk factors for MDRO and the prevalence of cultures positive for MDRO. We could speculate that not all risk factors for MDRO should be equally weighted and share the same impact on guiding empiric antibiotic therapy in sepsis.

Among healthcare-related risk factors, hospitalization in the previous 90 days is the strongest independent variable associated with MDRO-related sepsis. The increasing prevalence of MDRO within the hospital wards, due to an extensive antibiotic use and transmission between healthcare workers and patients, might explain this finding, as previously suggested in published manuscripts [15, 19, 21, 26, 32]. Also, septic shock is a risk factor for sepsis caused by MDRO, although some could argue that septic shock is a marker of disease severity and should not be considered a risk factor itself. As previously reported, markers of disease severity were included as risk factors for MDRO mainly because of the impact that this finding might have in the clinical management. Our finding on septic shock as independent risk factor for MDRO clearly identifies a subgroup of more fragile patients who might deserve a broad-spectrum empiric antibiotic course based on their disease severity and risk of organ failure. In light of the high prevalence of MDRO we found and the high mortality rate of patients with septic shock, an antibiotic prescription for MDRO should be considered in shocked patients, especially if additional risk factors (e.g., previous hospitalization) are concomitant.

Among all MDRO, ESBL producer *Enterobacteriaceae* seems to be the most prevalent (9.7%), with ESBL producing *E. coli* and *K. pneumoniae* being 35% of all *E. coli* and *K. pneumoniae* isolated. These data are similar to those reported in the scientific literature: frequency of sepsis due to gram negative bacteria (GNB) is increasing worldwide and *E. coli* is the most frequent GNB found in septic patients admitted from the community [33–36]. The rate of ESBL production among *Enterobacteriaceae* varies from country to country but it is increasing through all Europe, with Italy having one of the highest prevalence [31]. We specifically identified that hospitalization in the previous 90 days is a specific risk factor for ESBL *Enterobacteriaceae*, showing the important role played by the contact with health care setting, and suggesting the necessity of administering carbapenems empirically in septic patients with this risk factor. The other risk factor associated with ESBL+ bacteria is a positive history of stroke which could be explained in light of the prolonged hospitalization, nursing home residency or use of indwelling invasive devices (e.g., nasogastric tube, gastrostomy tube, bladder catheter). Considering the prevalence of ESBL *Enterobacteriaceae* and in case a MDRO infection is suspected (e.g., previous hospitalization), an empiric antibiotic therapy including carbapenems for ESBL+ pathogens could be considered, while waiting for culture results.

Sepsis due to MDRO is associated with higher mortality rates and the isolation of a MDRO is an independent risk factor for mortality. We know that patients presenting with MDRO infection have often a high number of comorbidities and a longer medical history, but also that MDRO infection and an inappropriate empirical antibiotic therapy are greatly correlated one to the other [5, 6]. In some studies MDRO infection is an independent risk factor for mortality, whereas in others it is a risk factor for inappropriate antibiotic therapy being the last an independent risk factor for mortality [5, 15].

Our study has some limitations, including the retrospective nature and the single-center design. Our primary outcome was mortality due to all causes during hospitalization, while sepsis-related mortality and long term outcomes would have been additional and interesting primary outcomes. Finally, we found 41.7% of population who received an empiric antibiotic therapy not concordant with local guidelines. Our local guidelines suggest broad antimicrobial spectrum antibiotics in patients with a suspicion of MDRO infection according to a list of risk factors recorded from previously published literature. So far, no specific risk factors for single MDRO in septic patients were identified. For the first time, we showed hospitalization in the previous 90 days and stroke as independently associated with ESBL+ bacterial infection in sepsis. Finally, our results should be interpreted with caution and not be widely generalized, as different causative pathogens of sepsis, different rates of antibiotic resistances, as well as risk factors related to different healthcare organizations might be recognized worldwide.

The strength and novelty of our study lie on a specific analysis of all risk factors for MDRO in a large sample of consecutive patients coming from the community and admitted to the ED, during a 5-year period, with the diagnosis of sepsis according to Sepsis-3 definition [4]. Several studies evaluated risk factors for MDRO in either bacteremic patients or in those affected by single organ disease (e.g., pneumonia). Our study is the first one evaluating all risk factors for MDRO previously published in literature in patients with sepsis regardless the site of infection.

## Conclusion

In conclusion, our finding of an isolation of a MDRO in 17% of patients with sepsis coming from the community advocates for a better recognition of possible risk factors for MDRO and especially for ESBL+ *Enterobacteriaceae*. Patients with sepsis who have been hospitalized in the previous 90 days and/or with a history of stroke might be ideal candidate for a broader empiric antibiotic therapy covering ESBL+ *Enterobacteriaceae*, while waiting for microbiological results.

## Additional file

Additional file 1: **Table S1.** Local guidelines for empirical antibiotic therapy in sepsis and septic shock. **Table S2.** Characteristics of the study sample stratified by MDR bacterial infection. (518). **Table S3.** Characteristics of the study sample stratified by ESBL+ bacterial infection. (518). (DOCX 54 kb)

## Abbreviations

AIDS: Acquired immune deficiency syndrome
COPD: Chronic obstructive pulmonary disease
ED: Emergency department
ESBL: Extended-spectrum beta-lactamases
GNB: Gram negative bacteria
ICU: Intensive care unit
INR: International normalized ratio
IQR: Interquartile ranges
LOS: Length of hospital stay
LTCF: Long term care facility
MDR: Multi-drug resistant
MDRO: Multi-drug resistant organisms
MOF: Multi organ failure
MRSA: Methicillin-resistant Staphylococcus aureus
SOFA: Sequential Organ Failure Assessment
