# Supplementary material for: Prevalence, risk factors and outcomes of patients coming from the community with sepsis due to multidrug resistant bacteria
Source: Multidiscip Respir Med. 2019 Jul 5;14:23. doi: 10.1186/s40248-019-0185-4 (PMC6610920; doi:10.1186/s40248-019-0185-4)
Supplement: Supplementary file 1 — Table S1. Local guidelines for empirical antibiotic therapy in sepsis and septic shock. Table S2. Characteristics of the study sample stratified by MDR bacterial infection. (518). Table S3. Characteristics of the study sample stratified by ESBL+ bacterial infection. (518). (DOCX 54 kb) [file 40248_2019_185_MOESM1_ESM.docx]

**Table S1. Local guidelines for empirical antibiotic therapy in sepsis and septic shock.**

| **Site of infection** | **I line antibiotic therapy** | **II line antibiotic therapy** | **Beta-lactam allergy** | **Risk factors for Gram - MDR bacteria (ESBL+)** | **Risk factors for Gram + MDR bacteria (MRSA)** |
| --- | --- | --- | --- | --- | --- |
| Lungs | Ceftriaxone + azithromycin  Or  Amoxicillin-clavulanate + azithromicin | Levofloxacin | Levofloxacin | Piperacillin-tazobactam + levofloxacin  Or  Imipenem/ meropenem | Levofloxacin + vancomycin |
| Abdomen | Piperacillin-tazobactam | Metronidazole + ciprofloxacin | Metronidazole + ciprofloxacin | Imipenem/ meropenem | Piperacillin-tazobactam + vancomycin |
| Biliary tract | Piperacillin-tazobactam | Metronidazole + ceftriaxone | Metronidazole + ciprofloxacin | Imipenem/ meropenem | Piperacillin-tazobactam + vancomycin |
| Urinary tract | Piperacillin-tazobactam | Ciprofloxacin | Ciprofloxacin | Imipenem/ meropenem |  |
| Genital tract | Clindamycin + aminoglycosides | Piperacillin-tazobactam + doxycycline |  | Imipenem/ meropenem |  |
| Soft tissue | Piperacillin-tazobactam + clindamycin | Imipenem/ meropenem + clindamycin | Levofloxacin + clindamycin | Imipenem/ meropenem + clindamycin | Imipenem/ meropenem + vancomycin |
| Unknown origin | Piperacillin-tazobactam +/- vancomycin | Ciprofloxacin + aminoglycosides +/- vancomycin | Ciprofloxacin + aminoglycosides +/- vancomycin | Imipenem/ meropenem + vancomycin | Piperacillin-tazobactam + vancomycin |
| Risk factors for ESBL +:   - Antibiotic therapy in the past 90 days or more than 3 antibiotic therapies in the past 12 months - Previous isolation of ESBL + bacteria in the patient or in close contact individuals - Long term care facilities residency - Current hospitalization (more than 5 days) or previous hospitalization in the past 3 months - Immunosuppression | | | | | |
| Risk factors for MRSA:   - Indwelling intravascular catheters - Long term care facilities residency - Previous isolation of MRSA in the patient - Implant of prosthetic devices in the past 24 month - Current hospitalization (more than 5 days) or previous hospitalization in the past 3 months | | | | | |
| Risk factors for Pseudomonas aeruginosa:   - Bronchiectasis - COPD GOLD stage IV - Long term care facilities residency - Current hospitalization (more than 5 days) or previous hospitalization in the past 3 months | | | | | |

**COPD**: chronic obstructive pulmonary disease

**Table S2. Characteristics of the study sample stratified by MDR bacterial infection. (518)**

| **Variable** | **MDR-** | **MDR+** | **p-value** |
| --- | --- | --- | --- |
| *MDR pathogen isolated* | 430 (83.0) | 88 (17.0) | - |
| **Demographics characteristics** | | | |
| *Female, n (%)* | 179 (41.6) | 41 (56.6) | 0.39 |
| *Median (IQR) age* | 79 (70-86) | 78.5 (70.5-85.0) | 0.83 |
| **Comorbidities, n (%)** | | | |
| *COPD* | 93 (21.6) | 21 (23.9) | 0.65 |
| *Diabetes mellitus* | 107 (24.9) | 17 (19.3) | 0.27 |
| *Hypertension* | 246 (57.2) | 43 (48.9) | 0.15 |
| *Ischemic heart disease* | 89 (20.7) | 20 (22.7) | 0.67 |
| *Chronic heart failure* | 36 (8.4) | 14 (15.9) | 0.03 |
| *Peripheral vascular disease* | 24 (5.6) | 4 (4.6) | 1.0 |
| *Stroke* | 61 (14.2) | 22 (25.0) | 0.01 |
| *Hemiplegia* | 18 (4.2) | 3 (3.4) | 1.0 |
| *Dementia* | 59 (13.7) | 20 (22.7) | 0.03 |
| *Chronic liver disease* | 33 (7.7) | 5 (5.7) | 0.35 |
| *Cirrhosis* | 21 (4.9) | 4 (4.6) | 1.0 |
| *Chronic renal failure* | 76 (17.7) | 20 (22.7) | 0.27 |
| *Active dialysis* | 12 (2.8) | 4 (4.6) | 0.33 |
| *Solid cancer* | 57 (13.3) | 16 (18.2) | 0.22 |
| *Hematological malignancy* | 32 (7.4) | 13 (14.8) | 0.03 |
| *AIDS* | 3 (0.7) | 0 (0.0) | 1.0 |
| *Chemotherapy* | 21 (4.9) | 7 (8.0) | 0.25 |
| *Severe immunosuppression* | 77 (17.9) | 21 (23.9) | 0.19 |
| *Mild/moderate immunosuppression* | 65 (15.1) | 18 (20.5) | 0.21 |
| *Chronic steroid therapy* | 49 (11.4) | 19 (21.6) | 0.01 |
| *Median (IQR) Charlson comorbidity index* | 6 (4-8) | 7 (5-8) | 0.21 |
| **Risk factors, n (%)** | | | |
| *LTCF* | 33 (7.7) | 12 (13.6) | 0.07 |
| *Antibiotic therapy in the past 90 days* | 60 (14.0) | 20 (23.3) | 0.03 |
| *Hospitalization in the past 90 days* | 103 (24.0) | 44 (50.0) | <0.0001 |
| *Home wound care/infusion therapy* | 60 (14.0) | 22 (25.0) | 0.01 |
| *Day hospital attendance* | 81 (18.8) | 19 (21.6) | 0.55 |
| *Indwelling bladder catheter* | 50 (11.6) | 20 (22.7) | 0.006 |
| *Indwelling intravascular catheters* | 24 (5.6) | 7 (8.0) | 0.39 |
| **Clinical findings** | | | |
| *Median (IQR) body temperature, °C* | 38 (37.1-38.7) | 38.2 (37.2-38.6) | 0.59 |
| *Median (IQR) systolic blood pressure, mmHg* | 110 (90-135) | 110 (85-130) | 0.29 |
| *Median (IQR) diastolic blood pressure, mmHg* | 60 (50-70) | 60 (50-70) | 0.31 |
| *Median (IQR) mean blood pressure, mmHg* | 77 (63-93) | 76 (62-90) | 0.33 |
| *Median (IQR) heart rate, bpm* | 104 (88-120) | 100 (83-120) | 0.20 |
| *Median (IQR) oxygen saturation, %* | 94 (91-97) | 95 (93-98) | 0.10 |
| *Median (IQR) respiratory rate, bpm* | 22 (18-30) | 22 (18-30) | 0.91 |
| *Median (IQR) shock index* | 0.9 (0.7-1.2) | 1.0 (0.8-1.2) | 0.84 |
|  | | | |
| *Median (IQR) arterial pH* | 7.5 (7.4-7.5) | 4.5 (7.4-7.5) | 0.26 |
| *Median (IQR) PaCO_2_, mmHg* | 30 (25-35) | 31 (26-36) | 0.13 |
| *Median (IQR) PaO_2_, mmHg* | 65.0 (55.0-76.5) | 67 (56.0-81.0) | 0.50 |
| *Median (IQR) HCO_3_- mEq/L* | 21.6 (17.8-24.3) | 22.0 (18.0-25.0) | 0.27 |
| *Median (IQR) PaO_2_/FiO_2_ ratio* | 276 (229-329) | 278.5 (233.0-343.0) | 0.34 |
| *Median (IQR) lactate, mEq/L* | 2.9 (2.1-4.5) | 2.6 (1.5-3.6) | 0.02 |
| *Median (IQR) white blood cells, cell/L^-1^* | 12.1 (7.3-17.8) | 12.0 (6.9-19.3) | 0.76 |
| *Median (IQR) platelet, cell/L^-1^* | 188.0 (132.5-255.0) | 202.0 (106.5-285.5) | 0.90 |
| *Mean (SD) haemoglobin, g/dL* | 12.5 (2.3) | 11.5 (2.2) | 0.0003 |
| *Median (IQR) glucose, mg/dL* | 144 (112-198) | 120.5 (101.0-162.0) | 0.002 |
| *Median (IQR) urea, mg/dL* | 65 (45-95) | 68.0 (48.5-109.5) | 0.34 |
| *Median (IQR) creatinine, mg/dL* | 1.6 (1.2-2.3) | 1.7 (1.2-2.9) | 0.30 |
| *Median (IQR) C-reactive protein, g/dL* | 12.0 (4.8-23.1) | 9.1 (3.7-19.0) | 0.11 |
| *Median (IQR) Aspartate aminotransferase* | 29 (20-54) | 24.5 (16.0-36.0) | 0.007 |
| *Median (IQR) Alanine aminotransferase* | 23 (14-40) | 17.0 (9.5-27.5) | 0.001 |
| *Median (IQR) total bilirubin, mg/dL* | 0.8 (0.5-1.8) | 0.8 (0.6-1.3) | 0.90 |
| *Median (IQR) INR* | 1.3 (1.2-1.5) | 1.3 (1.2-1.5) | 0.98 |
| **Site of infection, n (%)** | | | |
| *Lung* | 236 (62.9) | 37 (45.7) | 0.004 |
| *Urinary tract* | 123 (32.8) | 43 (53.1) | 0.001 |
| *Central nervous system* | 8 (2.1) | 1 (1.2) | 1.0 |
| *Abdomen* | 48 (12.8) | 11 (13.6) | 0.85 |
| *Skin and soft tissue* | 24 (6.4) | 7 (8.6) | 0.47 |
| *Bone and joints* | 4 (1.1) | 0 (0.0) | 1.0 |
| *Multiple origin* | 65 (15.1) | 17 (19.3) | 0.33 |
| *Unknown origin* | 55 (12.8) | 7 (8.0) | 0.20 |
| **Severity of disease, n (%)** | | | |
| *Hemodynamic failure* | 159 (37.3) | 41 (47.1) | 0.09 |
| *Respiratory failure* | 79 (18.6) | 15 (17.2) | 0.76 |
| *Renal failure* | 187 (43.9) | 36 (42.4) | 0.79 |
| *Liver failure* | 60 (14.9) | 7 (8.9) | 0.16 |
| *Cognitive impairment* | 130 (32.4) | 28 (35.0) | 0.65 |
| *Haematological dysfunction* | 42 (9.8) | 9 (10.6) | 0.82 |
| *Coagulation dysfunction* | 50 (12.9) | 6 (7.9) | 0.22 |
| *Metabolic dysfunction* | 301 (77.0) | 58 (69.1) | 0.13 |
| *Shock* | 109 (25.4) | 37 (42.1) | 0.002 |
| **Microbiological findings, n (%)** | | | |
| *Blood cultures performed in the first 48 h* | 373 (86.7) | 73 (83.0) | 0.35 |
| *Bacteraemia* | 144 (38.7) | 54 (74.0) | <0.001 |
| *Polymicrobial infection* | 15 (6.9) | 14 (15.9) | 0.01 |
| *ESBL producer pathogen isolated* | 0 (0.0) | 50 (56.8) | <0.0001 |
| *MRSA isolated* | 0 (0.0) | 20 (22.7) | <0.0001 |
| *Appropriate empiric antibiotic therapy according to guidelines* | 227 (59.1) | 46 (54.8) | 0.46 |
| *Use of vasopressors* | 67 (15.6) | 17 (19.3) | 0.39 |
| *Mechanical ventilation* | 8 (2.9) | 0 (0.0) | 0.37 |
| *Deaths* | 98 (23.1) | 35 (40.2) | 0.001 |

**n**: number; **IQR**: interquartile range; **COPD**: chronic obstructive pulmonary disease; **AIDS**: Acquired immune deficiency syndrome; **LTCF**: long term care facility; **INR**: International normalized ratio; **MOF**: multi organ failure (other than primary site of infection).

**Table S3. Characteristics of the study sample stratified by ESBL+ bacterial infection. (518)**

| **Variable** | **ESBL-** | **ESBL+** | **p-value** |
| --- | --- | --- | --- |
| *ESBL producer pathogen isolated, n (%)* | 468 (90.4) | 50 (6.7) | - |
| **Demographics characteristics** | | | |
| *Female, n (%)* | 199 (42.5) | 21 (42.0) | 0.94 |
| *Median (IQR) age* | 79.0 (69.5-86.0) | 79.5 (75.0-86.0) | 0.21 |
| **Comorbidities, n (%)** | | | |
| *COPD* | 106 (22.7) | 8 (16.0) | 0.28 |
| *Diabetes mellitus* | 110 (23.5) | 14 (28.0) | 0.48 |
| *Hypertension* | 264 (56.4) | 25 (50.0) | 0.39 |
| *Ischemic heart disease* | 97 (20.7) | 12 (24.0) | 0.59 |
| *Chronic heart failure* | 41 (8.8) | 9 (18.0) | 0.04 |
| *Peripheral vascular disease* | 25 (5.3) | 3 (6.0) | 0.76 |
| *Stroke* | 68 (14.5) | 15 (30.0) | 0.005 |
| *Hemiplegia* | 18 (3.9) | 3 (6.0) | 0.44 |
| *Dementia* | 65 (13.9) | 14 (28.0) | 0.008 |
| *Chronic liver disease* | 36 (7.7) | 2 (4.0) | 0.57 |
| *Cirrhosis* | 22 (4.7) | 3 (6.0) | 0.72 |
| *Chronic renal failure* | 88 (18.8) | 8 (16.0) | 0.63 |
| *Active dialysis* | 14 (3.0) | 2 (4.0) | 0.66 |
| *Solid cancer* | 62 (13.3) | 11 (22.0) | 0.09 |
| *Hematological malignancy* | 37 (7.9) | 8 (16.0) | 0.053 |
| *AIDS* | 3 (0.64) | 0 (0.0) | 1.0 |
| *Chemotherapy* | 24 (5.1) | 4 (8.0) | 0.33 |
| *Severe immunosuppression* | 86 (18.4) | 12 (24.0) | 0.33 |
| *Mild/moderate immunosuppression* | 73 (15.6) | 10 (20.0) | 0.42 |
| *Chronic steroid therapy* | 58 (12.4) | 10 (14.7) | 0.13 |
| *Median (IQR) Charlson comorbidity index* | 6.0 (4.0-8.0) | 7.0 (5.0-8.5) | 0.02 |
| **Risk factors, n (%)** | | | |
| *LTCF* | 38 (8.1) | 7 (14.0) | 0.16 |
| *Antibiotic therapy in the past 90 days* | 69 (14.8) | 11 (22.5) | 0.16 |
| *Hospitalization in the past 90 days* | 124 (26.5) | 23 (46.0) | 0.004 |
| *Home wound care/infusion therapy* | 70 (15.0) | 12 (24.0) | 0.10 |
| *Day hospital attendance* | 92 (16.7) | 8 (16.0) | 0.53 |
| *Indwelling bladder catheter* | 57 (12.2) | 13 (26.0) | 0.007 |
| *Indwelling intravascular catheters* | 28 (6.0) | 3 (6.0) | 1.0 |
| **Clinical findings** | | | |
| *Median (IQR) body temperature, °C* | 38.0 (37.1-38.7) | 37.9 (37.0-38.6) | 0.70 |
| *Median (IQR) systolic blood pressure, mmHg* | 110 (90-135) | 110 (90-125) | 0.73 |
| *Median (IQR) diastolic blood pressure, mmHg* | 60 (50-70) | 60 (50-70) | 0.76 |
| *Median (IQR) mean blood pressure, mmHg* | 77.0 (63.0-93.0) | 76.5 (63.0-90.0) | 0.76 |
| *Median (IQR) heart rate, bpm* | 103 (88-120) | 96 (80-120) | 0.13 |
| *Median (IQR) oxygen saturation, %* | 94 (91-97) | 96.0 (93.0-97.5) | 0.07 |
| *Median (IQR) respiratory rate, bpm* | 22 (18-30) | 20.0 (18.5-31.0) | 0.70 |
| *Median (IQR) shock index* | 0.9 (0.7-1.2) | 0.9 (0.8-1.1) | 0.54 |
|  | | | |
| *Median (IQR) arterial pH* | 7.5 (7.5-7.5) | 7.5 (7.4-7.5) | 0.38 |
| *Median (IQR) PaCO_2_, mmHg* | 30 (25-35) | 30.0 (26.0-35.5) | 0.65 |
| *Median (IQR) PaO_2_, mmHg* | 65 (55-77) | 63 (55-82) | 0.73 |
| *Median (IQR) HCO_3_- mEq/L* | 21.8 (17.9-24.5) | 22.0 (18.0-25.5) | 0.49 |
| *Median (IQR) PaO_2_/FiO_2_ ratio* | 277.5 (229.0-333.0) | 273.5 (233.0-338.0) | 0.86 |
| *Median (IQR) lactate, mEq/L* | 2.9 (2.1-4.4) | 2.7 (1.4-3.4) | 0.16 |
| *Median (IQR) white blood cells, cell/L^-1^* | 12.3 (7.6-18.2) | 8.9 (6.0-16.8) | 0.10 |
| *Median (IQR) platelet, cell/L^-1^* | 191.5 (133.0-259.0) | 182.0 (98.0-243.0) | 0.40 |
| *Mean (SD) haemoglobin, g/dL* | 12.4 (2.3) | 11.5 (2.2) | 0.01 |
| *Median (IQR) glucose, mg/dL* | 142.0 (110.0-196.5) | 129.0 (107.0-168.0) | 0.12 |
| *Median (IQR) urea, mg/dL* | 66 (46-99) | 62.5 (45.0-88.0) | 0.39 |
| *Median (IQR) creatinine, mg/dL* | 1.6 (1.2-2.4) | 1.5 (1.1-2.6) | 0.50 |
| *Median (IQR) C-reactive protein, g/dL* | 12.0 (4.8-23.4) | 7.2 (2.7-17.0) | 0.007 |
| *Median (IQR) Aspartate aminotransferase* | 28.5 (20.0-50.0) | 26.0 (18.0-37.5) | 0.36 |
| *Median (IQR) Alanine aminotransferase* | 22 (13-39) | 17 (10-35) | 0.24 |
| *Median (IQR) total bilirubin, mg/dL* | 0.8 (0.5-1.6) | 0.8 (0.7-1.4) | 0.92 |
| *Median (IQR) INR* | 1.3 (1.2-1.5) | 1.3 (1.1-1.4) | 0.48 |
| **Site of infection, n (%)** | | | |
| *Lung* | 256 (62.6) | 17 (36.2) | <0.0001 |
| *Urinary tract* | 138 (33.7) | 28 (59.6) | <0.0001 |
| *Central nervous system* | 8 (2.0) | 1 (2.1) | 1.0 |
| *Abdomen* | 53 (13.0) | 6 (12.8) | 0.97 |
| *Skin and soft tissue* | 28 (6.9) | 3 (6.4) | 1.0 |
| *Bone and joints* | 4 (1.0) | 0 (0.0) | 1.0 |
| *Multiple origin* | 75 (16.0) | 7 (14.0) | 0.71 |
| *Unknown origin* | 59 (12.6) | 3 (6.0) | 0.25 |
| **Severity of disease, n (%)** | | | |
| *Hemodynamic failure* | 181 (39.0) | 19 (38.8) | 0.98 |
| *Respiratory failure* | 84 (18.2) | 10 (20.0) | 0.76 |
| *Renal failure* | 207 (44.9) | 16 (32.0) | 0.08 |
| *Liver failure* | 63 (14.4) | 4 (8.9) | 0.37 |
| *Cognitive impairment* | 141 (32.5) | 17 (36.2) | 0.61 |
| *Haematological dysfunction* | 44 (9.4) | 7 (14.6) | 0.25 |
| *Coagulation dysfunction* | 53 (12.6) | 3 (7.3) | 0.45 |
| *Metabolic dysfunction* | 324 (75.9) | 35 (72.9) | 0.65 |
| *Shock* | 126 (26.9) | 20 (40.0) | 0.05 |
| **Microbiological findings, n (%)** | | | |
| *Blood cultures performed in the first 48 h* | 403 (86.1) | 43 (86.0) | 0.98 |
| *Bacteraemia* | 167 (41.5) | 31 (72.1) | <0.0001 |
| *Polymicrobial infection* | 25 (9.7) | 4 (8.0) | 1.0 |
| *MDR pathogen isolated* | 38 (8.1) | 50 (100.0) | <0.0001 |
| *MRSA isolated* | 18 (3.9) | 2 (4.0) | 1.0 |
| *Appropriate empiric antibiotic therapy according to local guidelines* | 249 (59.4) | 24 (49.0) | 0.16 |
| *Use of vasopressors* | 76 (16.2) | 8 (16.0) | 0.96 |
| *Mechanical ventilation* | 8 (2.7) | 0 (0.0) | 1.0 |
| *Death* | 117 (25.3) | 16 (32) | 0.27 |

**n**: number; **IQR**: interquartile range; **COPD**: chronic obstructive pulmonary disease; **AIDS**: Acquired immune deficiency syndrome; **LTCF**: long term care facility; **INR**: International normalized ratio; **MOF**: multi organ failure (other than primary site of infection).
